# Reversible spin storage in metal oxide—fullerene heterojunctions

**DOI:** 10.1126/sciadv.aax1085

**Published:** 2020-03-20

**Authors:** T. Moorsom, M. Rogers, I. Scivetti, S. Bandaru, G. Teobaldi, M. Valvidares, M. Flokstra, S. Lee, R. Stewart, T. Prokscha, P. Gargiani, N. Alosaimi, G. Stefanou, M. Ali, F. Al Ma’Mari, G. Burnell, B. J. Hickey, O. Cespedes

**Affiliations:** 1 School of Physics and Astronomy, University of Leeds, Leeds LS2 9JT, UK.; 2 Stephenson Institute for Renewable Energy, Department of Chemistry, University of Liverpool, Liverpool L69 3BX, England.; 3 Beijing Computational Science Research Centre, 100193 Beijing, China.; 4 ALBA Synchrotron Light Source, E-08290 Barcelona, Spain.; 5 School of Physics and Astronomy, SUPA, University of St Andrews, St Andrews KY16 9SS, UK.; 6 Laboratory for Muon Spin Spectroscopy, Paul Scherrer Institute, 5232 Villigen, Switzerland.; 7 Department of Physics, Sultan Qaboos University, P.O. Box 36, 123 Muscat, Oman.

## Abstract

We show that hybrid MnO_x_/C_60_ heterojunctions can be used to design a storage device for spin-polarized charge: a spin capacitor. Hybridization at the carbon-metal oxide interface leads to spin-polarized charge trapping after an applied voltage or photocurrent. Strong electronic structure changes, including a 1-eV energy shift and spin polarization in the C_60_ lowest unoccupied molecular orbital, are then revealed by x-ray absorption spectroscopy, in agreement with density functional theory simulations. Muon spin spectroscopy measurements give further independent evidence of local spin ordering and magnetic moments optically/electronically stored at the heterojunctions. These spin-polarized states dissipate when shorting the electrodes. The spin storage decay time is controlled by magnetic ordering at the interface, leading to coherence times of seconds to hours even at room temperature.

## Teaser

Hybrid interfaces form a spin capacitor for the generation and storage of spin angular momentum via optical or electronic stimuli.

## INTRODUCTION

Promising platforms for future quantum technologies make use not only of the charge of electrons but also their spin angular momentum. This allows the design of storage media controlled by spin polarization and devices for low-power electronics operated via spin currents with no Joule heating, adding as well functionalities for sensing, mechanical, heat, and voltage converters, and in optoelectronics (*1*–*8*). Carbon-based molecules are of interest because of the small spin-orbit coupling of light elements and a lack of hyperfine interaction in ^12^C, leading to long spin coherence and diffusion times—the interval before an electron spin changes its direction. However, as far back as 2011, it was realized that molecular spintronics faced challenges when replicating effects exported from conventional crystalline devices due to bad reproducibility, low carrier mobility, and degradation (*9*). Nevertheless, molecular spintronics has remained a fruitful field of research because of the various novel behaviors and effects, unique to molecular systems and, in particular, interfaces, that can be exploited in multifunctional devices (*3*, *9*, *10*). Over the last decade, there has been a concerted effort to comprehend the complex spin-dependent charge interactions between molecules and metals, in particular the formation of spin-polarized interfaces and tunable surface states, where molecular materials offer unique behavior and tunability that can be exploited to produce multifunctional devices via charge transfer and hybridization when in contact with other materials (*11*–*15*). The possibility of storing spin angular momentum at room temperature over long timescales in scalable, stable, and optoelectrically controllable devices would open new paths of research in memories, computing, and quantum processing (*16*–*19*). For example, the effect could be used to write and delete information with light or electrical bias or as a rechargeable source of spin-polarized currents.

## RESULTS AND DISCUSSION

Here, we demonstrate the use of hybrid devices with C_60_/MnO_x_ interfaces charged with a magnetic electrode to store spin-polarized charge in stable, localized states. Charge accumulation and extraction are controlled by the spin-dependent density of states (DOS) of the interface (Materials and Methods and figs. S1 and S2) (*20*). Because of charge transfer and relatively high electron mobility, C_60_ behaves as an n-type semiconductor with long spin diffusion length and highly efficient spin-electrical conversion (*21*–*25*). Molecules are weakly bound via van der Waals forces. When in contact with a metal, the C_60_ lowest unoccupied molecular orbitals (LUMOs) overlap to create a narrow conduction band just above the Fermi energy (*26*). At the interface between C_60_ and a metal, hybridization leads to a broadening of the LUMO and narrowing of the band gap, resulting in conducting surface states (*27*). These hybrid interface states can be spin split, leading to spin polarization and emergent magnetism (*11*, *13*, *28*–*31*).

**Fig. 1:**
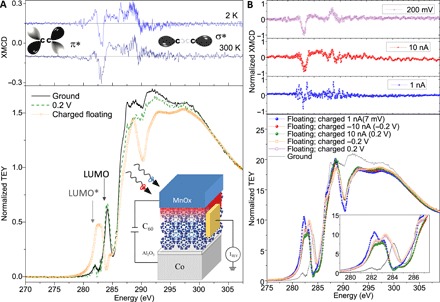
Spin-polarized charge trapping. (**A**) Bottom: NEXAFS at the carbon K-edge in a SiO_2_//Co(30)/Al_2_O_3_(1.6)/ C_60_(15)/MnO_x_(2.5) junction—film thickness in brackets in nanometers. After a bias is applied and then removed, the LUMO broadens and shifts to 283 eV. This persists until the electrodes are connected to a common ground. Top: π* LUMO states extend up to ~290 eV. In this region, XMCD measurements in the charged state after a bias show spin polarization at room and low temperature. (**B**) Changes in the carbon K-edge (bottom) and XMCD (top) with the applied voltage or current. XMCD is measured in the floating charged state. The different impedance of the source meter leads to changes in the trapped charge during the measurement and in the LUMO* peak. The higher internal impedance of the voltage source results in better signal-to-noise XMCD measurements.

We use near-edge x-ray absorption fine structure (NEXAFS) measurements in the total electron yield mode (TEY) to probe the carbon K-edge of C_60_ before, during, and after an applied voltage bias in Co/Al_2_O_3_/C_60_/MnO_x_ junctions (Fig. 1) (*32*, *33*). Before a bias, the K-edge shows the LUMO peak at 284 eV (*13*, *34*). With 0.2 V across the junction, the NEXAFS spectrum is largely unaffected. While the voltage is applied, there is a current flow across the interface. The trap states are continuously populated and depopulated, because their energy is small compared with the electrical bias, preventing the charging of the interface and generating scattering, spin depolarization, and noise in the NEXAFS signal. Once the voltage is removed and the device charged with the electrodes floating, the 284 eV peak is moved to 283 eV, which we will refer to as LUMO*. This change in the edge structure is stable over hours during NEXAFS measurements. X-ray magnetic circular dichroism (XMCD) reveals a polarization dependence around the LUMO* and π* orbitals in the charged state at room and low temperatures (Fig. 1, top) (*35*, *36*). Changes in the TEY magnitude can also be observed at higher energies. However, these states remain nonpolarized, and the peak position does not change. The original NEXAFS signal with the LUMO peak and no dichroism is recovered by draining the accumulated charge to a common ground. The magnetic dichroism is only present for the charged LUMO*, so it is not a consequence of spin-polarized hybrid interface states. The effect is dependent on the magnitude of the electrical bias and current density (Fig. 1B). The direction of the applied current/voltage does not change the result, as the trap states are charged using either polarity.

**Fig. 2:**
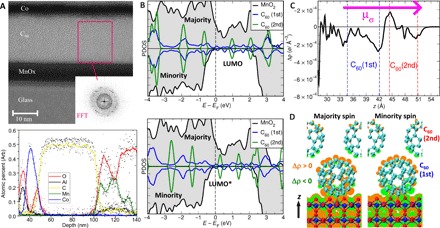
Interface modeling. (**A**) Top: High-resolution cross-sectional TEM image of a Co(5)/C_60_(20)/MnO_x_(10) junction. Inset: Fast Fourier transform (FFT) of the region indicated shows peaks in the diffraction pattern due to nanocrystalline grains in the sublimed C_60_ film. Bottom: Elemental chemical analysis of the interface—note the Gaussian profile of the e-beam (~10 nm full width at half maximum). The oxygen-to-manganese ratio is ~2:1 close to the C_60_ interface. (**B**) Spin-polarized density of states (PDOS) in the neutral (top) and with 0.6 *e*^−^ added to the C_60_ interface (bottom). The formation of a C_60_─O bond at the surface leads to a strong interfacial dipole and the generation of half-metallic interface states. (**C**) Changes in the charge density with respect to the neutral system along the direction perpendicular to the interface plane (*z*). The profile reveals the formation of a dipole layer density (μ_σ_) owing to accumulation of negative charge at the C_60_/β-MnO_2_(110) junction, and positive charge on the opposite side of C_60_(1st) toward C_60_(2nd). (**D**) Computed change in total charge density after 0.6 *e*^−^ are added to the C_60_ interface (|Δρ| = 10^−6^ *e* Å^−3^) for the oxidized, chemically bound minima for spin-up and spin-down states.

Cross-sectional transmission electron microscopy (TEM) images show clean interfaces and short-range order in the C_60_ film (Fig. 2A)—confirmed by a (111) face-centered cubic (FCC) peak in x-ray diffraction (fig. S2) (*20*, *37*). TEM images show different contrast across the MnO_x_ layer. Chemical analysis evidences stoichiometric changes, with an oxygen-rich C_60_ interface (Mn:O ratio of 1:2) and a more metallic core (Fig. 2A). β-MnO_2_ is the equilibrium phase of MnO_2_ at standard pressure and temperature (*38*). Density functional theory (DFT) simulations of C_60_/β-MnO_2_(110)-2x2 show half-metallicity, with the Fermi energy for minority spin electrons lying in an empty region of the DOS and with conducting states for majority spin electrons (Fig. 2B, top, and figs. S3 to S6) (*39*). The first C_60_ layer in contact with the oxide C_60_(1st) is conducting and spin polarized, whereas the second layer C_60_(2nd) is van der Waals bonded to the first and thus retains its bulk electronic structure, with a gap separating the localized highest occupied molecular orbital and LUMO states. In TEY, the signal from the film decays exponentially with the critical exponent given by the electron escape depth, so the LUMO signal is dominated by C_60_(2nd), with little contribution from the deeper layers. Our devices have a typical capacitance of the order of nanofarad under voltages of 0.1 V. This is equivalent to ~0.5 to 1 *e*^−^ per interface cage. Upon addition of 0.6 *e*^−^ to the metallic interface states (table S2) (*20*), DFT simulations show that the molecular orbital states for the C_60_(2nd) layer are downshifted in energy by 0.5 to 1 eV (Fig. 2B, bottom), in good agreement with the measured LUMO to LUMO* shift (Fig. 1). Surface oxygen preferentially bonds to C_60_, creating a strong interfacial dipole and rectifying barrier (Fig. 2C). The LUMO shift takes place due to the formation of this dipole, although 98% of the stored charge is localized at the C_60_(1st) layer. DFT calculated spin-resolved charge density maps when 0.6 *e*^−^ charge is added to the interface, with larger trapping of minority spin electrons at the C_60_ metal oxide sites, are shown in Fig. 2D. The magnetic moment of C_60_(1st) in the neutral configuration is 0.05 μ_B_ per cage, increasing to 0.18 μ_B_ once the interface is charged. The moment in C_60_(2nd) is 0.0 μ_B_ before and after charging.

**Fig. 3:**
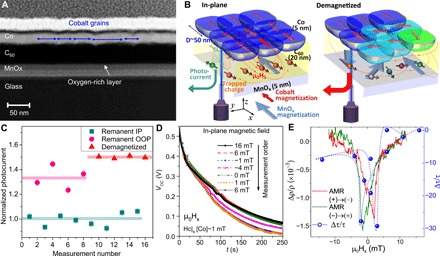
Spin stabilization facilitated by stray fields. (**A**) Wide area TEM image showing the presence of grains some ~50 to 100 nm in lateral size. (**B**) Schematic for the stray field μ_o_H_s_ generated by Co grains and the buildup of spin-polarized trapped charge via photocurrents. (**C**) Photocurrent in Co(5)/C_60_(20)/MnO_x_(10) devices as a function of the magnetic history. Lower remanent magnetization leads to less efficient charge trapping and therefore a higher photocurrent. (**D**) Discharge of the open-circuit voltage (*V*_OC_) under a series of in-plane magnetic fields μ_o_H_x_. The discharge time τ is up 30% faster at the coercive field, when the magnetization of the cobalt film is nil. (**E**) The magnetic field dependence of τ follows the anisotropic magnetoresistance (AMR) of the Co electrode, showing the correlation between Co magnetization and spin-polarized charge storage (blue line is a guide to the eye).

Wide-field TEM images show that the sputtered Co layer is polycrystalline, with each grain several tens of nanometers in size (Fig. 3A). Although ideal, uniformly magnetized two-dimensional films have a nil demagnetization factor and no stray field, there will be magnetization discontinuities at these grain boundaries. These stray fields lead, e.g., to muon spin depolarization in the vicinity of a ferromagnetic film. In the MnO_x_ layer, the magnetism is confined to the surface, and junctions with a CuO_x_ electrode replacing MnO_x_ do not show dichroism (figs. S7 and S8) (*20*). If the long spin coherence time of trapped charge measured via XMCD is linked to the stabilization of minority spins via stray fields from Co grains, introducing magnetic disorder in the Co layer will break the spin alignment and reduce charge trapping (Fig. 3B). We test this by measuring photocurrents generated in MnO_x_(5)/C_60_(20)/Co(t) junctions at different magnetic states: in remanence after a saturation in-plane field (remanent magnetization M_r_ ~90% of saturation, M_s_), after an out-of-plane (OOP) field (M_r_ ~0.2 M_s_), and after demagnetizing the film with an oscillating damped field (M_r_ ~0) (fig. S9) (*7*, *20*). When M~M_s_, the stray field is uniform and stabilizes the spins in the opposite direction to the Co and MnO_x_ magnetization. The minority spin charge is trapped at the interface due to a lack of available spin-down states to diffuse into, reducing the photocurrent. When the electrodes are disordered, the stray field does not necessarily stabilize the spin direction opposite to the MnO_x_ magnetization, and charges abandon the traps via thermal fluctuations—the energy depth of the traps being of the order of tens of megaelectron volts (fig. S10) (*20*). The photocurrent is then increased in this magnetically disordered state by up to 50% (Fig. 3C). MnO_2_ is exchange split only at the surface, with a magnetic moment that is orders of magnitude smaller than the thicker Co electrode. Furthermore, the magnetization in MnO_2_ is limited to the surface with a negligible magnetocrystalline anisotropy, so no stray fields are expected.

Once the light is removed and the charged device is in a floating state, the junction behaves as a leaky capacitor where the accumulated charge, stabilized by the interfacial dipole, moves into the oxide electrode in a time scale dependent on the capacitance of the device and the spin polarization of the trapped charge: τ = *C*/*G*, with *C* (*G*) the spin-dependent capacitance (conductance) of the interface. Charge diffusion to the cobalt electrode may be possible, but electrons would need to hop across several tens of C_60_ layers and, in some samples, tunnel through an Al_2_O_3_ barrier to reach the metal contact. The discharge time τ is also dependent on the magnetic structure. When the Co electrode is disordered (e.g., at the in-plane coercive field), charge drains more efficiently. This leads to a 20 to 30% faster voltage drop than when the electrode magnetization is uniform (saturated in plane) (see Fig. 3, D and E).

**Fig. 4:**
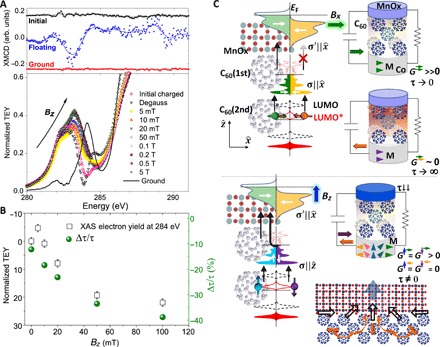
Magnetic field effects and schematic of the trapped spin capacitor mechanism. (**A**) Top: XMCD for the initial, charged (floating), and discharged (ground) states in a Co/Al_2_O_3_/C_60_/MnO_x_ junction showing spin-polarized trapped charge. Bottom: NEXAFS in remanence after OOP magnetic fields (B_z_). The 283 eV LUMO* peak is displaced toward the pristine LUMO until the Co electrode is magnetically saturated OOP. (**B**) Decay time constant τ for optically charged junctions, typically ~30 to 300 s for 100 × 100 μm^2^ junctions (fig. S11) (*35*). The dependence of τ on H_z_ is similar to that of the TEY at the pristine 284 eV LUMO position. (**C**) Schematic for spin capacitance. Top: Minority electrons are trapped when the quantization axis C_60_ layer (σ) is parallel to that of the C_60_/MnO_x_ interface (σ′). Bottom: When σ ≠ σ′, the conductivity *G* is above zero for both spins. In particular, for σ ⊥ σ′, both spin orientations in C_60_(2nd) transfer to the interface majority band with the same probability and conductivity (≠0).

The polarization of the trapped charge is coupled to the magnetization of the cobalt electrode. Applying increasingly higher OOP fields and measuring at remanence lead to domain formation and demagnetization. The disordered stray fields and changes in magnetization cause the charge traps to vacate, and the LUMO* peak shifts toward the pristine LUMO (Fig. 4A). This LUMO* displacement can be correlated with changes in the discharge time after a junction is optically charged. At remanence after an OOP field, the interface is partly depolarized and τ drops by ~40% (Fig. 4B). The change in τ is similar to the one seen at the in-plane coercive field, since both maximize the magnetic disorder by bringing the magnetization to zero.

Minority spins at the interface layer, C_60_(1st), are localized. They cannot move to the electrode unless they undergo spin flip, as there are no minority states available at the Fermi level of the MnO_x_ interface for minority spin electrons to tunnel into. In a charged device, the zero-bias conductance can be approximated by Fermi’s golden rule (*40*)

$$G=\sum_{\sigma \sigma^{\prime }}G^{\sigma \sigma^{\prime }}(E_{F})=\frac{2\pi e^{2}}{\hbar }\sum_{\sigma \sigma^{\prime }}{|T_{21}^{\sigma \sigma^{\prime }}|}^{2}\rho_{2}^{\sigma }(E_{F})\rho_{1}^{\sigma^{\prime }}(E_{F})$$

Where G^σσ^′^^(*E*_F_) is the spin-dependent conductance, $T_{21}^{\sigma \sigma \prime }$ is the transmission matrix element for an electron with spin σ from C_60_ tunneling to the spin σ^′^ band in the MnO_x_ interface, and $\rho_{2}^{\sigma^{\prime }}(E_{F})\;[\rho_{1}^{\sigma }(E_{F})]$ is the spin split DOS in the initial C_60_ (interface) state. The tunneling probability depends on the projection of the trapped spin at the initial C_60_ layer onto the quantization axis of the final layer. If σ and σ^′^ are quantized in the same axis, due to spin conservation, then *G*^⇄^ = *G*^⇆^ = 0. Since there are no available states at the interface minority band (e.g., σ^′^←), G^⇇^ = 0. However, there are available states for majority electrons, so *G* = *G*^⇉^≠0. When Co is disordered, stray fields with different orientations are generated, and the quantization axis σ and σ^′^are not well defined nor parallel across the device, leading to G ≠ 0 for both orientations (Fig. 4C).

**Fig. 5:**
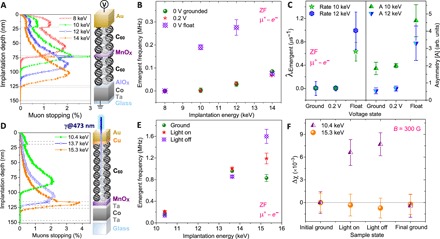
LE-μSR detection of local magnetic moments in charged samples. (**A**) Calculated penetration profiles for the voltage-biased sample (*46*). (**B**) The μ^+^@C_60_[−] frequency is increased in the charged state at 10 and 12 keV due to the enhanced local magnetization at the C_60_/MnO_x_ interfaces when charged. (**C**) Increased μ^+^@C_60_[−] depolarization rate and oscillation amplitude in the charged state at 10 and 12 keV. (**D**) Penetration profiles for the photovoltaic sample. (**E**) The μ^+^@C_60_[−] frequency is higher during the photocurrent (light on) and increases further in the charged floating state (light off). (**F**) Changes in magnetic susceptibility due to spin-polarized trapped charge at the C_60_/MnO_x_ interface (15.3 keV) are absent in the bulk C_60_ film (10.4 keV).

To confirm the presence of localized magnetic ordering due to optical or electrical charging, low-energy muon spin rotation (LE-μSR) was used. In LE-μSR, a beam of almost fully polarized positive muons (μ^+^) is accelerated at kilo–electron volt energies to be implanted 10 to 100 nm into a sample, providing a highly sensitive probe of the local magnetization (*41*, *42*). Muon acceleration voltages are applied at the moderator, away from the sample, to avoid charging effects. A sample with the structure Co(20)/Al_2_O_3_(2)/C_60_|_bottom_(50)/MnO_x_(3)/C_60_|_top_(50)/Au(25) is charged with the same 0.2 V potential used in NEXAFS (Fig. 5A). Measurements are taken at 250 K, where molecules rotate on timescales (~ps) much faster than the muon decay (~μs). It is then possible to detect the precession in zero magnetic field (ZF) assigned to charged C_60_[−] (*11*, *29*). This state is formed in proximity with metals or when the C_60_ layer is charged, leading to lower cage symmetry and an anisotropic hyperfine coupling of the endohedral muonium state (μ^+^@C_60_[−]), with a precession frequency of 0.2 to 1.5 MHz—similar to that observed in ellipsoidal C_70_ (*43*–*45*). A zero frequency shows the absence of C_60_[−] away from cobalt (8 to 10 keV) in the ground or biased states. As in NEXAFS, the μSR signal is unchanged during an applied voltage, but the μ^+^@C_60_[−] frequency, depolarization, and asymmetry (oscillation amplitude) close to the MnO_x_ interface (10 to 12 keV) are increased in a charged, floating state—all indicative of local magnetism. We attribute the changes to Zeeman splitting of the anisotropic hyperfine levels for μ^+^@C_60_[−] in the magnetic field generated by the trapped polarized charge (Fig. 5, B and C).

A second sample with the structure Ta(5)/Co(20)/Ta(5)/MnO_x_(10)/C_60_(100)/Cu(5)/Au(15) is photovoltaic. A blue laser is used to generate a photocurrent of 125 μA and charge the interface without a bias (Fig. 5D). The cobalt electrode is close to the active MnO_x_/C_60_ interface but spin decoupled via a tantalum layer. Measurements in ZF show again an increase in the μ^+^@C_60_[−] frequency during (no bias to discharge the states during the measurement) and after light irradiation (Fig. 5E). The direct current (DC) local magnetic susceptibility is calculated from the diamagnetic response in the muon precession frequency during an applied transverse field of 300 G. The susceptibility increases during and after light irradiation, but only for the energy probing the interface, and reverses to the normal value upon grounding (Fig. 5F and figs. S12 to S15) (*39*). No light irradiation effect is observed in LE-μSR measurements of samples without an MnO_x_ interface.

## CONCLUSION

The combination of stray fields, spin-polarized interfacial dipole formation, and interface-limited transport observed in MnO_x_/C_60_ provides a mechanism for spin-dependent charge trapping via an electric bias or light irradiation. The available states and discharge period can be controlled via magnetic fields and remain stable at room temperature for macroscopic timescales, leading to optically and/or electrically generated local magnetism. This effect opens new paths of research for electro-optic manipulation of spin information and the development of spin capacitors.

## MATERIALS AND METHODS

Junctions were fabricated in a combination sputtering/evaporation chamber using shadow masks to create a vertical junction with a crossed electrode configuration. Metal electrodes were deposited using DC magnetron sputtering in an Ar atmosphere at a pressure of ~2 × 10^−6^ mbar, while the molecular layer was thermally evaporated without breaking vacuum at a pressure of 10^−8^ mbar. MnO_x_ was deposited via sputtering deposition from a 99% pure Mn target and plasma oxidized in situ. Intercalation of molecular oxygen into C_60_ crystals may lead to a breakdown of the fullerenes into graphitic remnants and carbon monoxide at 470 K. However, intercalated oxygen has been observed to desorb from bulk crystals at only 450 K. C_60_ structures can be grown in the same chamber as complex oxides, provided the crucible is below 450 K in the presence of oxygen. Manganese oxide was deposited in a multistep sputtering process. First, metallic manganese was sputtered from a 99% pure target in an argon atmosphere. The manganese electrode was then plasma oxidized in an oxygen/argon atmosphere at a 5:1 ratio. For photovoltaic measurements, aluminosilicate glass was used as a transparent, inert, insulating, and amorphous substrate. C_60_ was evaporated onto the manganese oxide electrode to form a 20-nm-thick molecular film. The second electrode was sputtered directly onto the C_60_ film. While sputtering is a fairly aggressive technique, the mechanical strength and crystalline structure of C_60_ films allow for sputtered films of light metals without denaturing the molecules or allowing significant interdiffusion. This is confirmed in cross-sectional TEM, which shows minimal interdiffusion between cobalt and C_60_.

## Supplementary Material

aax1085_SM.pdf

## SUPPLEMENTARY MATERIALS

Supplementary material for this article is available at http://advances.sciencemag.org/cgi/content/full/6/12/eaax1085/DC1 Section S1. Sample structure Section S2. Junction characterization Section S3. DFT simulations Section S4. X-ray absorption spectroscopy Section S5. Sample preparation and LE-μSR fitting details Fig. S1. Transport characteristics of junctions with and without an alumina barrier. Fig. S2. Thin film characterization. Fig. S3. DFT geometries. Fig. S4. DFT optimized geometries for the neutral and charged states. Fig. S5. PDOS calculations. Fig. S6. Band structure and molecular orbitals around the Fermi energy. Fig. S7. NEXAFS experimental details. Fig. S8. NEXAFS and XMCD when using a CuO_x_ electrode. Fig. S9. Photovoltaic effect in C_60_/MnO_x_ junctions. Fig. S10. Dependence of the photocurrent of the LE-μSR sample with temperature. Fig. S11. Discharge for a photovoltaic-charged device. Fig. S12. LEM experimental details for electrically charged sample. Fig. S13. Fitting of LEM data for electrically charged sample. Fig. S14. LE-μSR probing of a photovoltaic sample at 50 K in a transverse field of 300 G. Fig. S15. Magnetometry data. Table S1. Computed energy differences (eV) between the different adsorption geometries of C_60_ on β-MnO_2_(110)-2x2 as a function of the extra electronic charge added to the system. Table S2. Computed Bader charges (Q) (*63*) for the different adsorption geometries of C_60_ on β-MnO_2_(110)-2x2 as a function of the extra electronic charge added to the system. References (*47*–*67*)
