# Supplementary material for: Reversible spin storage in metal oxide—fullerene heterojunctions
Source: Sci Adv. 2020 Mar 20;6(12):eaax1085. doi: 10.1126/sciadv.aax1085 (PMC7083605; doi:10.1126/sciadv.aax1085)
Supplement: aax1085_SM.pdf [file aax1085_SM.pdf]

Science Advances
AAAS

advances.sciencemag.org/cgi/content/full/6/12/eaax1085/DC1

Supplementary Materials for

Reversible spin storage in metal oxide—fullerene heterojunctions

T. Moorsom, M. Rogers, I. Scivetti, S. Bandaru, G. Teobaldi, M. Valvidares, M. Flokstra, S. Lee, R. Stewart, T. Prokscha, P. Gargiani, N. Alosaimi, G. Stefanou, M. Ali, F. Al Ma'Mari, G. Burnell, B. J. Hickey, O. Cespedes*

*Corresponding author. Email: o.cespedes@leeds.ac.uk

Published 20 March 2020, *Sci. Adv.* **6**, eaax1085 (2020)
DOI: 10.1126/sciadv.aax1085

This PDF file includes:

Section S1. Sample structure
Section S2. Junction characterization
Section S3. DFT simulations
Section S4. X-ray absorption spectroscopy
Section S5. Sample preparation and LE-μSR fitting details
Fig. S1. Transport characteristics of junctions with and without an alumina barrier.
Fig. S2. Thin film characterization.
Fig. S3. DFT geometries.
Fig. S4. DFT optimized geometries for the neutral and charged states.
Fig. S5. PDOS calculations.
Fig. S6. Band structure and molecular orbitals around the Fermi energy.
Fig. S7. NEXAFS experimental details.
Fig. S8. NEXAFS and XMCD when using a CuO_x electrode.
Fig. S9. Photovoltaic effect in C_{60}/MnO_x junctions.
Fig. S10. Dependence of the photocurrent of the LE-μSR sample with temperature.
Fig. S11. Discharge for a photovoltaic-charged device.
Fig. S12. LEM experimental details for electrically charged sample.
Fig. S13. Fitting of LEM data for electrically charged sample.
Fig. S14. LE-μSR probing of a photovoltaic sample at 50 K in a transverse field of 300 G.
Fig. S15. Magnetometry data.
Table S1. Computed energy differences (eV) between the different adsorption geometries of C_{60} on β-MnO_2(110)-2x2 as a function of the extra electronic charge added to the system.
Table S2. Computed Bader charges (Q) (*63*) for the different adsorption geometries of C_{60} on β-MnO_2(110)-2x2 as a function of the extra electronic charge added to the system.
References (*47–67*)

Section S1. Sample structure

Two characteristic structures for the NEXAFS/Photovoltaic junctions used in this research are: Co(10-30 nm)/ Al_2O_3(1.4 -1.6 nm)/C_{60}(20 nm)/MnOx(2.5-5 nm)/Al(1.2 nm) and Co(10-30 nm)/C_{60}(20 nm)/ MnOx(2.5-5 nm)/Al(1.2 nm) –with Cu instead of Co in some samples. The sputtering pressure is of the order of mTorr, but sputtering and evaporation are not done simultaneously. The C_{60} is evaporated at ~450 °C with a chamber pressure of ~10^{-8} mTorr and an oxygen pressure of ~10^{-10} mTorr. The use of a tunnel barrier determines whether there is spin conserved injection from the ferromagnetic electrode or whether spin scattering at the interface leads to a non-polarized current as well understood in the conductivity mismatch problem.(*47*) Whether or not there is a direct contact between metal and molecule also affects the doping of the C_{60} film. Where there is a direct metal contact, the C_{60} behaves as though it were significantly n-doped, making the IV characteristic more asymmetric.*(21)* With a tunnel barrier, the IV characteristic is more symmetric. In both cases, charge trapping is observed to occur at the MnOx/C_{60} interface. Assuming the modeled MnO_2/C_{60} interface is representative of the system in the real device, and based on the DFT simulations, only the immediate metal oxide/C_{60} interface has metallic states and is therefore the only conductive layer together with the Co-film. Deeper MnO_2 atomic-layers (3rd onwards), further than 0.5 nm (5 Å) from the interface, display a non-zero (>0.2 eV) band-gap. Based on the present DFT simulations, the role of the MnO_2 layer is to provide a source of O-atoms, whose diffusion, coupled with MnO_{2-x}/C_{60} re-hybridization and addition of extra charge in the floating state altogether lead to: 1) appearance of half-metallic states at the MnO_2/C_{60} interface (likely spin polarization but not half-metallicity in the real, disordered MnO_x system, leading to long but finite discharge times), and 2) spin-polarization of the C_{60} closest to the MnO_{2-x} substrate.

The cobalt film is polycrystalline, formed by grains of several 10s of nm. At remanence, these grains generate a stray field (even though an ideal, uniformly magnetized cobalt film would not). This stray field is a very common problem e.g. in our measurements of spin triplets with superconductors and muon spin spectroscopy. In the electrically-biased muon sample, we used the thickest C_{60} layer of any experiment, and the field at the interface, as measured by the muon precession, was 5 Gauss. The distance between Co and interface for this sample was 55 nm, compared to 15-20 nm in NEXAFS and photovoltaics, so the field in the latter samples can be estimated to be 100-200 Gauss assuming a dipolar field ($\propto r^{-3}$). This field is unlikely to be the only mechanism mediating the spin stabilization, which will depend as well on the spin dependent interfacial dipole and MnO_2 density of states, but it explains the connection between the Co magnetization and the interface transport properties (e.g. in the discharge time, the photocurrents measured with different Co configurations, the dependence of the LUMO* position on field etc.). By comparison, the MnOx film is only (weakly) magnetic at the atomic surface, with no magnetocrystallyne anisotropy (nominally no demagnetizing or stray field for the ideal 2D case) and therefore magnetically soft.

Section S2. Junction characterization

The interface has a resistivity orders of magnitude higher than the bulk MnOx layer, changing from 10 to 0.1 MΩ for a typical 100×100 μm^2 junction when the interface is broken by displacing oxygen with an electric field. For spectroscopy, XAS and XMCD, alumina barriers are used to prevent Co/C_{60} hybridization features appearing in the K-edge and to allow the injection of spin polarized electrons. For V_{OC} measurements, this tunnel barrier is not included. This is because the photovoltaic efficiency is much higher without a barrier, allowing us to

access a V_{OC} similar to the bias applied in XAS, and because the junction structure is inverted – figure S1. Inverting the structure allows us to excite optically the MnOx/C_{60} without a protective cap, thus increasing the illumination and eliminate artefacts from the cap used. The junction is charged by exposure to light and the generation of a photocurrent; we then measure the drop of the open circuit voltage with time by using a nano-voltmeter connected to a data acquisition card with sub-ms resolution. While oxygen diffusion is commonly observed at the interface between complex oxides and C_{60}, the process used to fabricate these samples shows minimal interdiffusion of oxygen in cross-sectional TEM.(*48*) This is vital since C_{60}-oxide develops a mid-gap acceptor band which would quench the interfacial effects and compensate donor transport, significantly reducing conductivity.(*49*)

Transport through the junctions is not uniform under low bias. A junction with an alumina tunnel barrier was probed via photo-luminescence spectroscopy during transport. Hot electron injection over the **A** nnel barrier creates secondary **C** itations and boosts the luminescent signal, highlighting areas where current density is highest.(*50*) This shows the importance of using optical excitation to measure the spin dependent leakage current, since charging of the interface during transport will occur preferentially at the junction edges where the stray fields are likely to behave differently to the junction center. Figures 2A-E shows characterization data for the C_{60} and MnO_x layers, including Xray reflectivity and diffraction (XRR/XRD), Raman spectroscopy and luminescence. Figure 2F shows the charge-trapping effects in the luminescence of C_{60}.

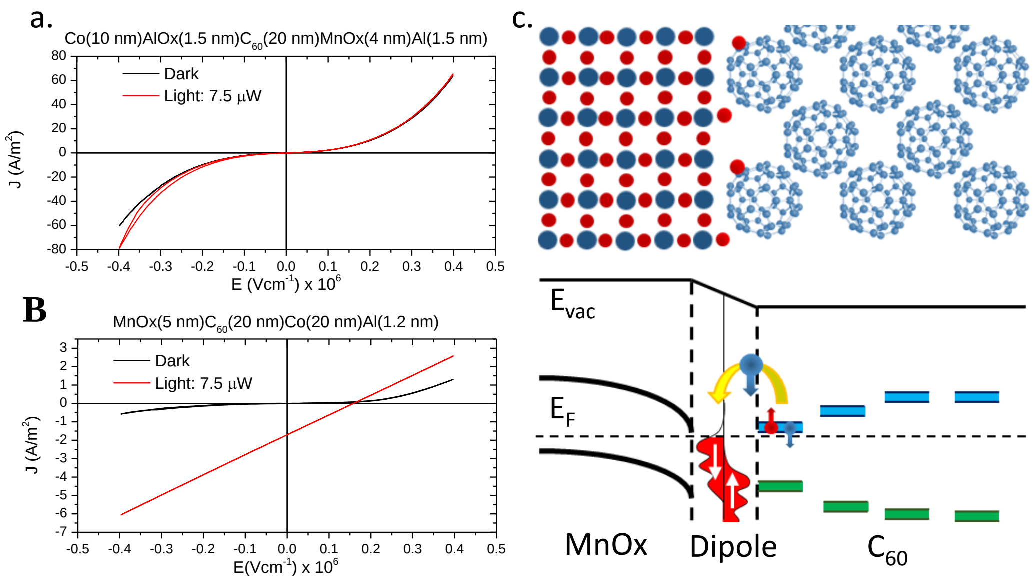

Fig. S1. Transport characteristics of junctions with and without an alumina barrier. (A) IV characteristic for a junction with an alumina barrier. This device has the same structure as the device used for XAS in figure 1. The barrier significantly reduces the efficiency of the photovoltaic interface such that VOC and short circuit current (ISC) are negligible. The lack of the contact barrier also means the total conductivity is higher. **(B)** Device with no alumina barrier. Here, the metal-molecule contact barrier and increased n-doping makes transport highly asymmetric and increases the efficiency of the photovoltaic effect - due to the interfacial electrical dipole and change in carrier mobility, electrons see a different effective barrier depending on the current polarity. **(C)** Illustration of the band structure at the MnOx/C_{60} interface. The MnOx behaves as a p-type semiconductor. At the interface, there is significant band bending due to the interfacial potential. This band bending and the presence of a large contact barrier is a common feature of crystalline-molecular semiconductor interfaces. At the interface, the C_{60}-O dipole layer creates a potential well which limits charge transport.(*51*) This interfacial model is supported by DFT and is similar to effects observed in LiF interfaces.(*52*)

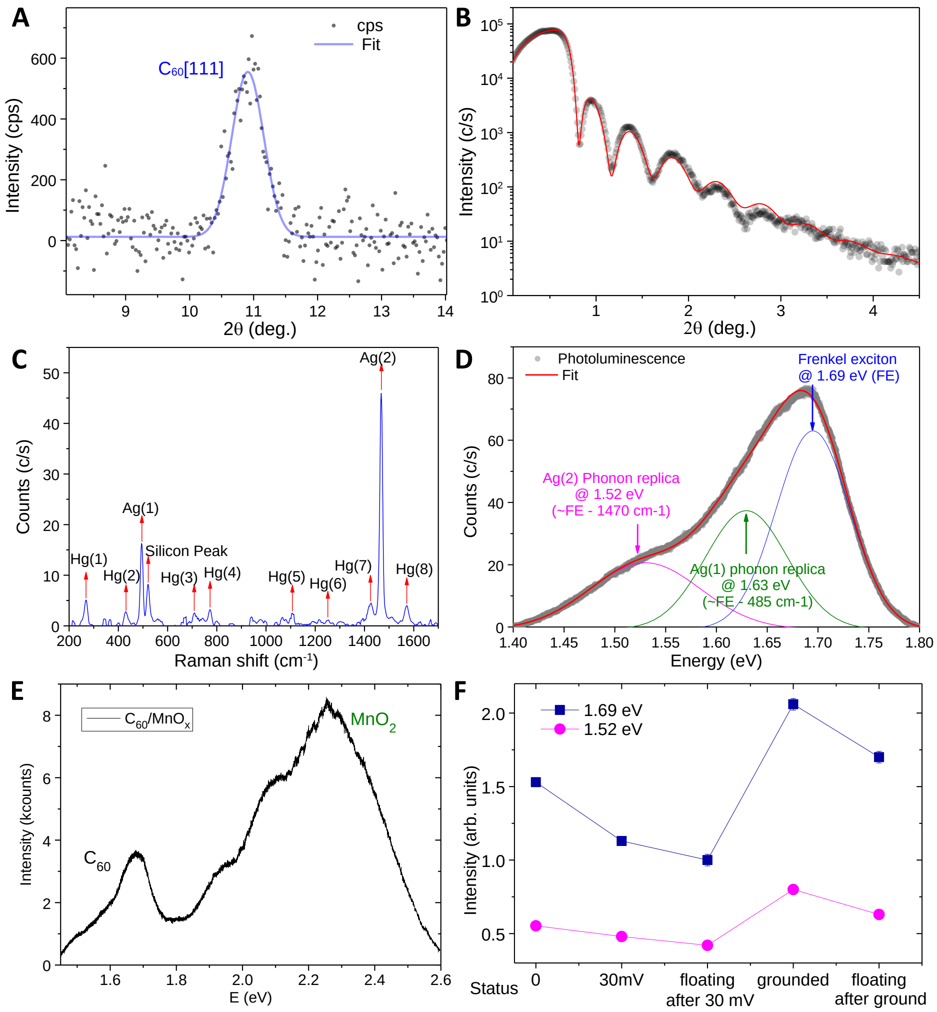

Fig. S2. Thin film characterization. A. XRD of a C_{60} layer showing the [111] FCC peak. **B.** XRR of MnO_x with a fit to Mn_2O_3(14 nm)/MnO_2(3 nm) –note the agreement in the critical edge, dependent on the density of the surface MnO_2 layer. **C.** Raman spectrum of a C_{60} film with the assigned vibrational modes. **D.** Luminescence spectrum of a C_{60} film with the main phonon replicas. **E.** Luminescence of a C_{60}/MnO_x bilayer excited at 473 nm (2.62 eV), with a Stokes shift of ~0.35 eV attributed to the β-MnO_2 gap. **F.** The luminescence due to Frenkel exciton (1.69 eV) and phonon-assisted recombination (1.52 eV) in C_{60} are reduced after an electric field is applied. The initial “0” state before a voltage is measured with the electrodes floating.

Section S3. DFT simulations

All the simulations presented in this work were spin-polarized and performed with the projector-augmented wave (PAW) method,(*53*) as implemented in the VASP code.(*54*-*56*) Following Refs. (*57*-*59*), anisotropic Hubbard corrections [DFT+(U-J)](*60*) were applied on the 3rd orbitals of the Mn atoms (U=6.1 eV, J=1.0 eV) to ameliorate limitations of the adopted PBE functional in describing manganese oxides.

The MnO_x substrate was modelled as a 2x2 (12.595 x 11.775 $Å^2$ in-plane periodicity), 4-layer slab of the energy-favored(*57*) β-MnO_2(110) surface. Following Ref. (*57*), the plane-wave kinetic cut-off was set to 400 eV and all the calculations were performed in a ferromagnetic spin configuration. The slab 2D Brillouin zone was sampled with 2x2 **k**-point grid defined according to the Monkhorst−Pack scheme.(*61*) Two layers of C_{60} were placed symmetrically on both side of the β-MnO_2(110)-2x2 slab to prevent the introduction of artificial dipoles in the simulations. In all cases, symmetry was switched off to allow full relaxation on the interfaces to within a force tolerance of 0.03 eV $Å^{-1}$, and van der Waals (vdW) corrections added as per formalism and parametrization proposed by Grimme.(*62*) Several adsorption geometries were explored by initially placing the 1st C_{60} layer at 3.5 Å [Fig. S3a], 2.0 Å [Fig. S3a] and 1.0 Å [Fig. S3c]. The rational for the latter case was to create a strongly repulsive initial geometry to model relaxation of the system in the presence of geometrical constraints, as likely present in the nm-corrugated real samples. In all cases at least 12 Å vacuum-buffer between replicated images was present along the direction perpendicular to the slab.

As shown in Fig. S3, several energy minima are possible for the adsorption of the first C_{60} layer [$C_{60}(1^{st})$] on β-MnO_2(110)-2x2. Notably, oxidation of the $C_{60}(1^{st})$ by diffusion of the topmost (bridging) O-atoms of β-MnO_2(110)-2x2 is energetically favored by 1.22 eV with respect to chemisorption of $C_{60}(1^{st})$ on a stoichiometric β-MnO_2(110) slab (Table S1). This result appears to be qualitatively unaffected by the presence of additional electronic charge at the C_{60}/β-MnO_2(110)-2x2 interface model (Table S1). In all cases, the non-perfectly matched periodicity of the β-MnO_2(110)-2x2 slab and the C_{60} layers resulted in asymmetric relaxation of the 2nd C_{60} layer [$C_{60}(2^{nd})$] over the $C_{60}(1^{st})$.

A B C

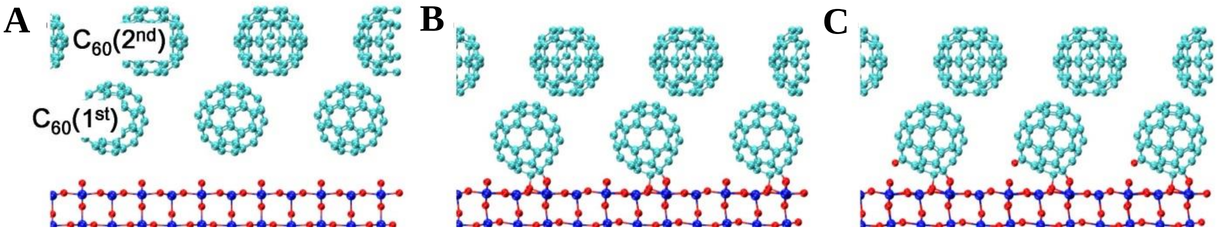

Fig. S3. DFT geometries. Side view of the optimized (neutral) C_{60}/β-MnO_2(110)-2x2 interface models. **(A)** vdW minimum (*vdW*). **(B)** chemically-bound minimum (*chem*). **(C)** oxidized chemically-bound minimum (*o-chem*). The relative energy of the systems decreases going from **A** (+3.8 eV) to **B** (+1.22 eV) to **C** (0 eV). That is, oxidation of the C_{60} by diffusion of the topmost (bridging) O-atoms of β-MnO_2(110) is energetically favored. Mn: blue; O: red; C: cyan.

Table S1. Computed energy differences (eV) between the different adsorption geometries of C_{60} on β-MnO_2(110)-2x2 as a function of the extra electronic charge added to the system. Owing to the presence of a compensating background for the charged systems, comparison between the results for different amount of extra charge is meaningless. "e^-" is defined as the elementary unit of charge.

**Geometry**	**neutral**	**+0.6 e^-**	**+1.2 e^-**	**+2.0 e^-**
vdw	+3.80 eV	//	//	//
chem	+1.22 eV	+1.79	+1.61	+0.26
o-chem	0.0	0.0	0.0	0.0

The progressive decrease of the energy difference between the *o-chem* and *chem* minima as a function of an increasing amount of extra charge added to the system stems from residual inaccuracies of the DFT+(U-J) method and the ensuing incapability of maintaining the extra charge at the intimate C_{60}/β-MnO_2(110)-2x2 interface without (artificial) charge delocalization on the 2nd C_{60} layer [Fig. S3] and Table S2. Owing to this undesirable aspect, the effects of extra charge in the C_{60}/β-MnO_2(110)-2x2 interface are analyzed only for addition of 0.6 e^- (0.3 e^-/C_{60}), which result in negligible erroneous charge-spillage on the 2nd C_{60} layer.

Table S2. Computed Bader charges (Q) (*63*) for the different adsorption geometries of C_{60} on β-MnO_2(110)-2x2 as a function of the extra electronic charge added to the system. The progressive charging of the 2nd C_{60} layer [C_{60}(2nd)] is due to limitation of the DFT+(U-J) corrections in preventing artificial delocalization of the extra charge. Accordingly, the ~0.01 e^- charge on C_{60}(2nd) for addition of 0.6 e^- in the system warrants for negligible delocalization errors in the simulations. Please note that there are **two** C_{60}(1st) and C_{60}(2nd) molecules per symmetric slab.

**System**	**Q(MnO2)**	**Q[C_{60}(1st)]**	**Q[C_{60}(2nd)]**	**Total Charge**
***chem* (neutral)**	-3.34	+1.67	**0.00**	0.00
***o-chem* (neutral)**	-4.56	+2.28	**0.00**	0.00
***chem* (+0.6 e^-)**	-3.45	+1.43	**-0.01**	-0.60
***o-chem* (+0.6 e^-)**	-4.83	+2.12	**-0.01**	-0.60
***chem* (+1.2 e^-)**	-3.72	+1.40	**-0.14**	-1.20
***o-chem* (+1.2 e^-)**	-5.10	+2.07	**-0.12**	-1.20
***chem* (+2.0 e^-)**	-4.00	+1.33	**-0.33**	-2.00
***o-chem* (+2.0 e^-)**	-5.12	+1.88	**-0.32**	-2.00

Although comparison between the energy favored *o-chem* geometries for the neutral and charged (+0.6 e^-) C_{60}/β-MnO_2(110)-2x2 interfaces indicates negligible changes owing to addition of the extra charge [Fig. S4], compelling evidence of the origin of the measured energy-shift for the LUMO level of the 2nd C_{60} layer is provided by electrostatic analysis of the interface.

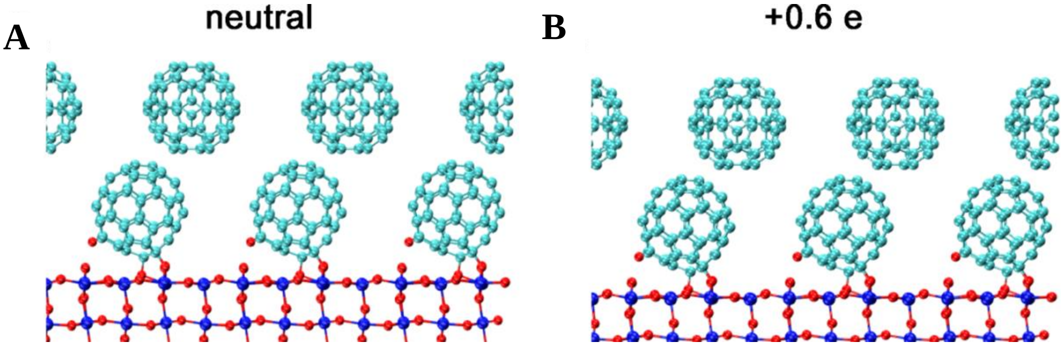

Fig. S4. DFT optimized geometries for the neutral and charged states. Side view of the optimized geometry for the neutral **(A)** and charged (+0.6 e, **B**) *o-chem* minima of the C_{60}/β-MnO_2(110)-2x2 interface. The computed changes in nuclear relaxation are evidently small. Mn: blue; O: red; C: cyan.

Fig. 2 in the manuscript displays the change in the average total (nuclear plus electronic) charge density, Δρ, induced by addition of 0.6 electrons to the neutral *o-chem* minimum and computed as

$$\Delta\rho = \rho(+0.6e^{-}) - \rho(neutral)$$

with the total charge density ρ obtained, via Poisson equation, from the curvature of the electrostatic potential (*64*) (and removal of the compensating background for the charged system).

For the specialist reader we finally note that, as shown in Fig. S5, re-hybridization between the C_{60} and β -MnO_2(110), in either the *chem* or *o-chem* minimum, turns out to be critical in removing metallic states for the minority spin-channel. When re-hybridization is not present as

for the *vdW* minimum, the β-MnO_2(110)-2x2 slab recovers a non-zero density of states for the spin-minority channel as previously computed for bare the β-MnO_2(110) at the same level of theory.(*57*)

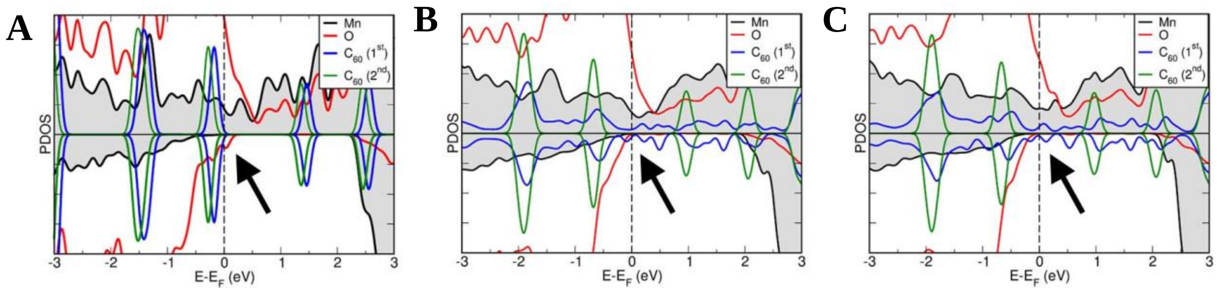

Fig. S5. PDOS calculations. Computed atom-projected Density of States (PDOS) for the *vdW* **(A)**, *chem* **(B)** and *o-chem* minima of the neutral C_{60}/β-MnO_2(110)-2x2 interface **(C)**. The black arrows mark the change in PDOS for the minority spin of the β-MnO_2(110) surface following re-hybridization with the C_{60} fullerene molecules.

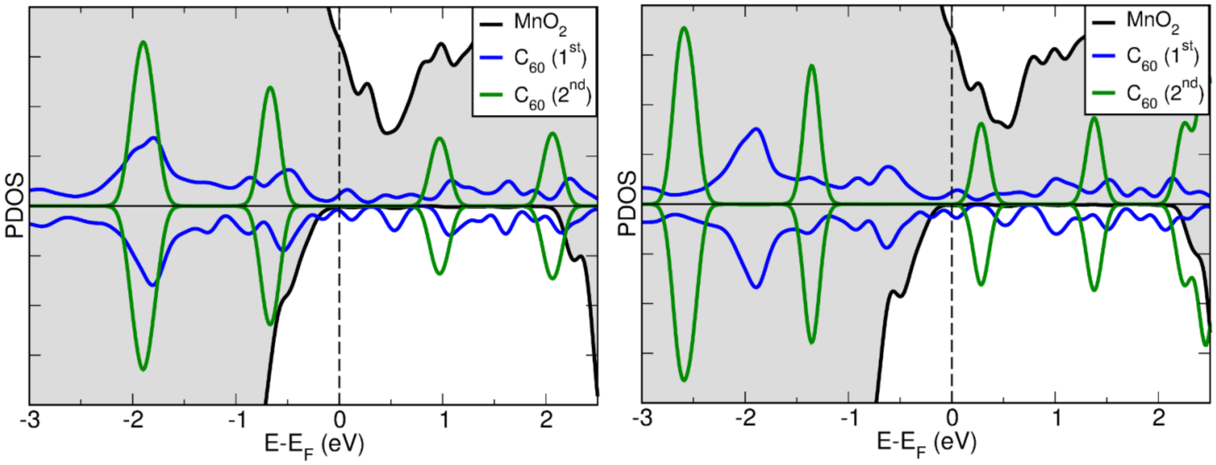

Fig. S6. Band structure and molecular orbitals around the Fermi energy. Zoom around the Fermi energy region of the PDOS for the neutral (left) and 0.6 e^-- charged interface (right).

Section S4. X-ray absorption spectroscopy

XAS studies were performed on junctions with the structures:

- Co(30nm)/Al_2O_3(1.4nm)/C_{60}(15nm)/MnOx(2.5nm)/Al(2nm)
- Cu(30nm)/Al_2O_3(1.4nm)/C_{60}(15nm)/MnOx(2.5nm)/Al(2nm)
- Co(30nm)/Al_2O_3(1.4nm)/C_{60}(15nm)/CuOx(2.5nm)/Al(2nm)

The active junction area for all samples in XAS was 100x100 µm. These samples were deposited on both SiO_2 and alumino-silicate glass substrates. XAS and XMCD spectra(data) were measured in the BOREAS beamline at the ALBA synchrotron near Barcelona.(*65*) The X-ray beam is produced by an elliptically polarized undulator and monochromatized by a variable line spacing grating monochromator (400 lines/mm at Carbon edge), with a total flux of the order of 10^{11} photons/s at the Carbon edge. The beam was collimated by variable focusing bender mirrors down to about 100 x 50 micron approximately, allowing the junction area to be accurately located via triangulation with the electrodes. The sample was measured in TEY mode using a Keithley 428 current amplifier with the drain contact made using silver coated carbon tape to a point close to the junction. Transport contacts are made using silver paint and kapton insulated Cu wire attached to sprung contacts built into the TEY head. The TEY drain contact is made using a thin strip of conducting carbon tape which extends to 1mm away from the junction and is then coated with silver. Contact is made between this tape and the Cu backing plate which is connected to ground via a pico-ammeter. The total resistance of this channel was measured to be 400 Ohm for the samples shown in Fig 1. The drain contact resistance was on the order of 100 Ω, and this similarity in resistance caused mixing between the drain current and junction current, increasing the signal to noise ratio. When there was no active bias, the internal resistance of the source was >1 GΩ.

Following monochromation, the beam is focused by a final mirror at which a photoelectron current is recorded (i1). The beam then passes through a gold grid at which the normalization signal is recorded (i2). Both the mirror and normalization grid have a small amount of carbon contamination resulting in absorption at 285 and 290 eV. The TEY signal is then normalized to the gold grid, figures S7A-C. To avoid distortion of the signal by carbon contamination on the normalization grid, fresh gold is evaporated onto the grid at the beginning of the experiment. Carbon contamination is a common issue so it is important to establish whether any component of the C K-edge arises from aliphatic or amorphous carbon layers rather than the C_{60} film. By probing carbon K-edge on the substrate away from the C_{60}, it was verified that the residual signal from carbon contaminants was much smaller than the C_{60} signal, Fig. S7B. The main XMCD peak at 282 eV features above any artefacts due to normalization. Owing to metallization and ensuing broadening of empty states, we do not expect a contribution to the (TEY) NEXAFs signal from $C_{60}(1^{st})$. By comparison, the $C_{60}(2^{nd})$ layer in the simulations has a well-defined band-gap and sharp LUMO(*) energies. In addition, deeper layers of C_{60}, beyond the 3rd layer (i.e. $\gtrsim$2 nm from the interface), will have an exponentially lower contribution toward the TEY signal, as the Auger electrons produced in these layers will not have enough energy to overcome the work function and contribute to the signal. Sum rules typically used in transition metals do not apply to the carbon K-edge, partly due to the lack of SOC and the orbital hybrid structure of carbon, so a priory it would be impossible to distinguish between spin or orbital momentum in C_{60} via XMCD. Therefore, we must indirectly infer the origin of the signal from other methods. Given that *i.* the effect emerges only when we use a magnetic electrode and a, theoretically, half-metallic interface; *ii.* that the muon depolarization responds to the local spin ordering; and *iii.* the spherical symmetry of the C_{60} cage (which rotates freely in the ps scale at room temperature in

thin films, which will average out any orbital asymmetry in the C_{60}), it is almost certain that the spin momentum is responsible for the results observed.

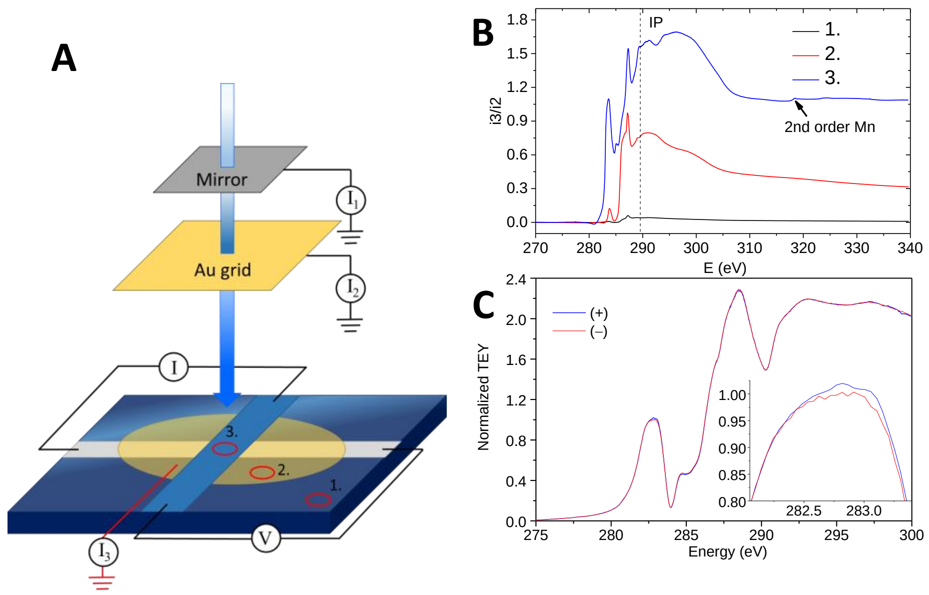

Fig. S7. NEXAFS experimental details. (A) Diagram showing the junction pattern with crossed electrodes of MnOx and Co separated by a C_{60} barrier. **(B)** The sample, scanned at three points, shows strong signals at point 3 and 2 where the K-edge here closely resembles control C_{60} films though modified under the MnOx electrode due to hybridization. That point 3 is in the junction center is confirmed by the 2nd order Mn excitation observed at 320 eV. At point 1, where there should be no C_{60}, a signal is detected resembling amorphous carbon but more than an order of magnitude weaker. This implies that carbon contaminants are negligible compared to the C_{60} signal. The dichroism features are the result of comparing the TEY signal at i3 for positive and negative circular polarizations and normalizing this difference to the difference between the TEY current from the gold calibration grid. **(C)** Comparison of the NEXAFS signal with clockwise/counter clockwise circular polarizations in the charged floating state.

Figure S8 is a comparison of changes in the NEXAFS and XMCD spectra of C_{60} junctions as a function of the top metal oxide.

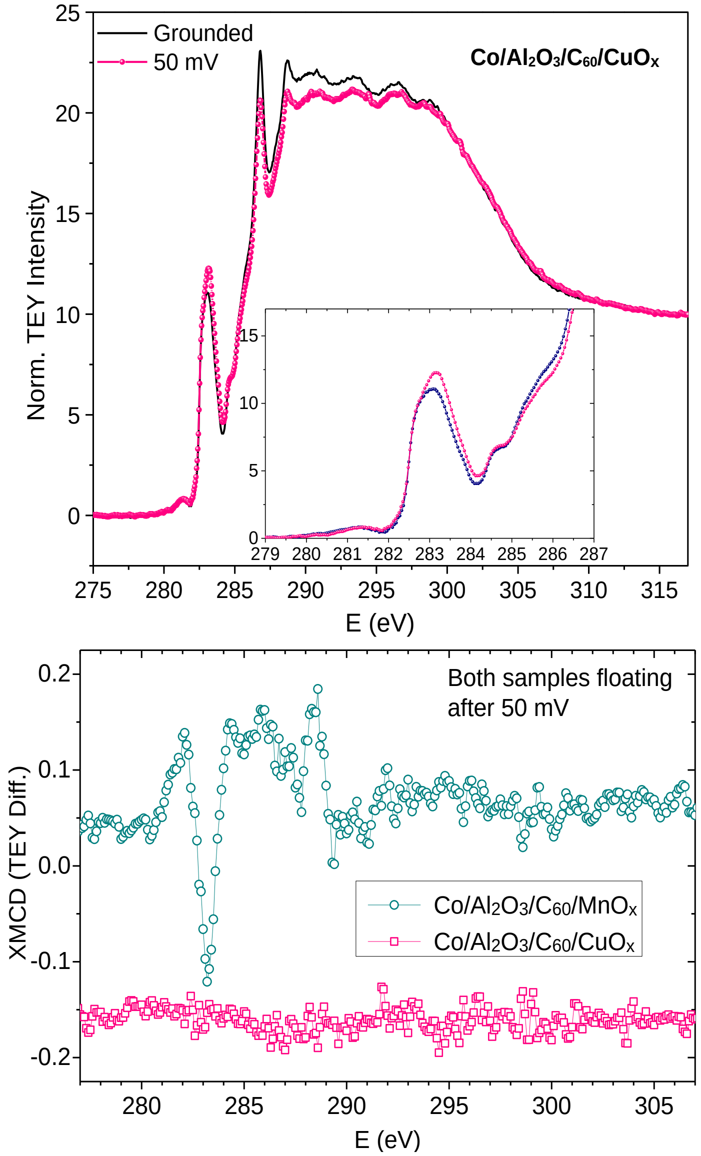

Fig. S8. NEXAFS and XMCD when using a CuO_x electrode. (Top) Change with an applied voltage for a C_{60}/CuOx junction. There are differences in the electron yield, but the position of the peaks remains mostly unchanged. Note that the TEY modifications are due to charging and not dichroism -both polarizations have been used in this figure. **(Bottom)** XMCD measurements after a 50 mV bias for a C_{60}/MnO_x and a C_{60}/CuO_x junction. There is no measurable dichroism in the copper sample.

The C_{60}/MnO_x interface acts as a pn junction with a weak photovoltaic response. Figure S9 shows the typical photovoltaic effect for a junction without an alumina barrier between the cobalt electron and C_{60} layer. The presence of an alumina barrier does not change this dependence, but greatly reduces the magnitude of the photocurrent.

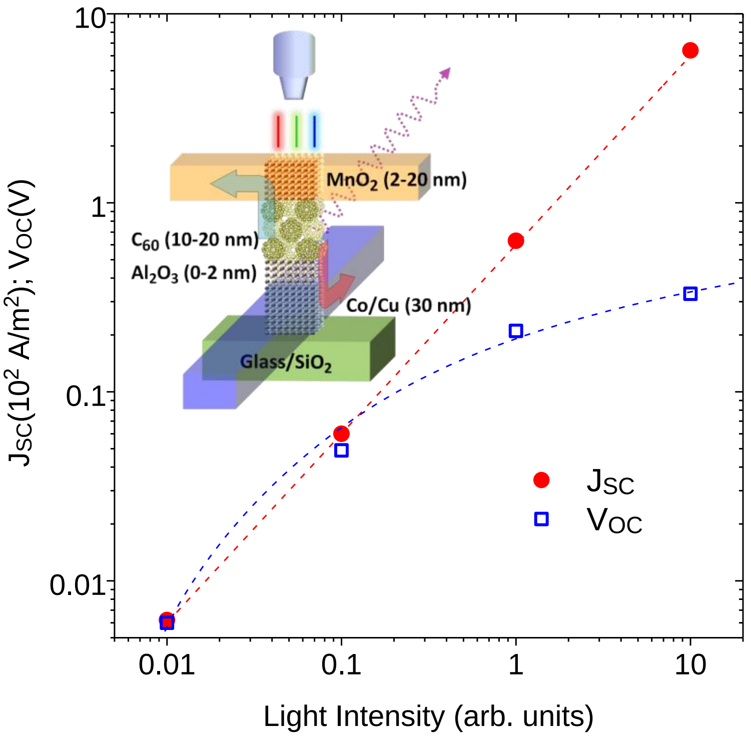

Fig. S9. Photovoltaic effect in C_{60}/MnO_x junctions. Typical photovoltaic response of a $MnO_x/C_{60}/Co$ 100x100 μm junction to optical irradiation at 473 nm with J_{SC} the short circuit current density and V_{OC} the open circuit voltage. Dashed lines are a linear fit of the current density to the light intensity and to $V_{OC} = \frac{nkT}{q} \ln\left(\frac{I_{SC}}{I_0} + 1\right)$, where n is the ideality factor (n=2.5 due to the high resistance of the manganese oxide electrode), k is Boltzmann's constant, T is the temperature, q the electron charge and I_0 the dark current (~100 pA). The inset schematic shows the setup for photoluminescence and photovoltaic measurements.

The photocurrent decreases as the temperature is lowered due to increased internal resistance and trap dwell time, with the carrier density and mobility reduced –see Figure S10.

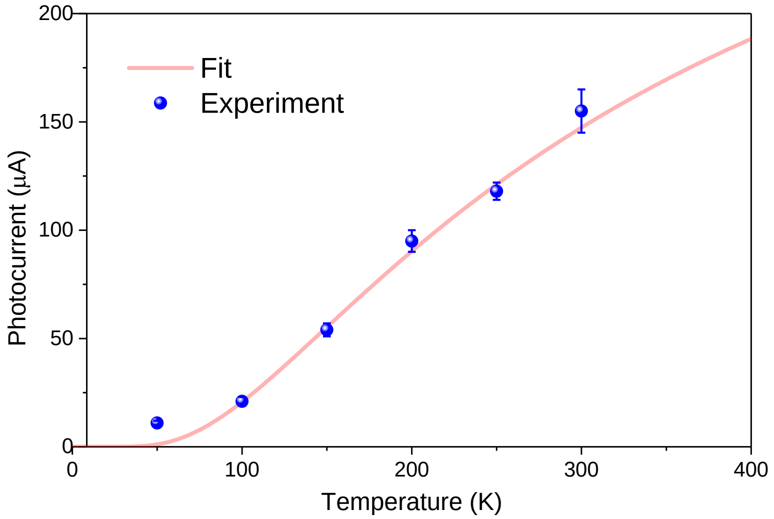

Fig. S10. Dependence of the photocurrent of the LE-μSR sample with temperature. The probability for an exciton to recombine depends on the dwell time on trap, and will therefore increase at lower temperatures –reducing the photocurrent.(*66*) The data can then be fit to the function $\mathrm{J} = \mathrm{J}_0 \exp\left(\frac{-\Delta}{\mathrm{kT}}\right)$ where $\Delta = 25 \pm 1$ meV is the trap depth and $\mathrm{J}_0 = 390 \pm 20$ μA is a factor that takes into account the charge carrier density, mobility and electric field.

Once the device is charged using the photovoltaic effect, stopping the light irradiation leads to a capacitor-like discharge where the hopping time, and therefore the discharge time, are dependent on the magnetic field. Magnetic configurations of the cobalt electrode with high disorder or a complex domain structure, e.g. measurements at the in-plane coercive field, see Figs. 3D-E of the main manuscript, or at remanence after an out-of-plane field (Fig. 4B and Fig. S11) have a decay 230-40% faster than when the electrode magnetization is uniform (in-plane saturated).

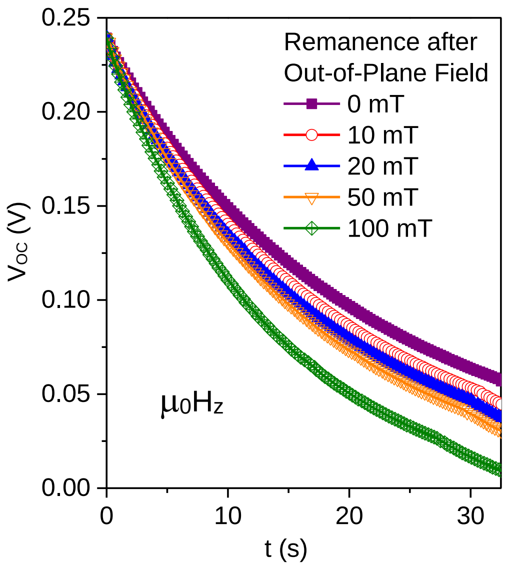

Fig. S11. Discharge for a photovoltaic-charged device. Changing the out-of-plane magnetic field leads to different discharge times when the device is measured at remanence; the larger fields, always below the out of plane saturation, lead to higher disorder.

Section S5. Sample preparation and LE-μSR fitting details

Low energy muon spin rotation (LE-μSR) measurements were taken at the LEM beamline, at the Paul Scherrer institute in Switzerland. Unlike bulk μSR, low energies of implantation may be obtained by use of a moderation technique.(*67*) The technique uses a few-hundred-nanometer thick solid Ar moderator capped with ~10nm N_2, grown on top of a 100μm thick silver foil. Implantation energies varying between 0.5 keV to 30 keV, corresponding to depths of up to ~200 nm, can be sourced by choosing appropriate transport and sample voltage settings –with the large voltages being applied to the muon beam away from the sample to avoid damage. The energies needed to probe the MnOx/C_{60} were chosen on the basis of the stopping profiles calculated using a Monte Carlo algorithm TRIM.SP.(*46*)

Data was collected in a zero field (ZF) and transverse field (TF) geometry (field in the plane of the sample and transverse to the spin of the incoming muons). In the TF geometry the initial polarization of the muon spin is perpendicular to the applied field. The time dependence of the muon polarization is analyzed as an asymmetry function $A_s(t)$ and data fitted using musrfit software. In zero field and at 250 K, the fast rotation of C_{60} molecules eliminates the observability of the anisotropic Mu-C_{60} radical state, therefore we only observe a low frequency (less than 1.5 MHz) oscillation attributable to transitions between different hyperfine energies of an axially symmetric, anisotropic endohedral muonium state (μ^+@C_{60}). Past measurements of weak magnetic states in C_{60} based interfaces show the coexistence of the endohedral muonium state with another that corresponds to the magnetic interfacial C_{60}, where the muonium states include particles in neutral C_{60} and in charged triplets, labelled as C_{60}[-] in the manuscript.*(9)* In our system, the stray field of the cobalt electrode strongly contributes to the depolarization of the muonium states.

LE-μSR requires large area sample due to long count times. Therefore, wide area molecular junctions, with roughly 200 mm^2 active areas, were fabricated with aid of optical lithography techniques. Firstly, Ta/Au contacts were deposited onto Corning Eagle XG substrates. The ferromagnetic electrode and tunnel barrier, Co(20nm)/Al_2O_3(2nm), was then deposited through a shadow mask. Here, the Al_2O_3 not only acts to protect the FM against Oxidation between fabrication steps, but will also become part of the tunnel barrier, ensuring spin conserved tunneling of charges from the Co into the molecular layer. To avoid short circuits, or current crowding due to variations in the electric field around the edge of the cobalt electrode, the active junction area was defined with an optical lithography step. A 200nm layer of PMMA A4950K

was spun onto the whole device. A 200 mm^2 window was then exposed to UV and ozone. This was finally developed in acetone and exposed to an Ar plasma to etch any residual PMMA and to leave a clean Al_2O_3 surface. The remainder of the device, starting with a second thin (1.4nm) Al_2O_3 layer, was deposited by sublimation of C_{60} and DC sputtering to yield wide area molecular junctions. A schematic of such a device is shown in fig. S12A, with the corresponding fraction of the muons stopping throughout the device. After using this fabrication processing, the DC I-V data shows a nonlinear dependence of the resistance with voltage. This is to be expected for transport dominated by tunneling. The resistance of the devices also increases at lower temperature, suggesting that after charge carriers tunnel into the C_{60} LUMO, undergo the expected variable-range hopping transport, see Fig. S12B for a typical I-V characteristic in these large-scale devices.

We first model the time dependence of the muonium polarization with a relatively simple model. A highly damped oscillation of the μ^+@C_{60} state is chosen with a depolarization function which incorporates slowly relaxing mechanisms –including in a single function the oscillation for both neutral and charged C_{60}. The resulting ZF-μSR spectra are fitted with the function

$$A_s(t) = A\cos(\varphi + 2\pi v_{\mu+@C60}t)e^{-\lambda_{\mu+@C60}*t} + A_{fast}e^{-\lambda_{fast}*t}$$

In this equation the frequency $v_{\mu+@C60}$ corresponds to hyperfine oscillations of any anisotropic endohedral muonium. For this model, it incorporates the typical frequency expected for muonium (μ^+-e-) and that of the muonium forming with interfacial/charged C_{60} triplets. The parameter A is the muon decay asymmetry parameter and is proportional to the volume occupied

by the corresponding μ+ state. The phase angle, φ is the angle of the muon spin at t=0 with respect to the positron detector. λ is a decay constant determined by the polarization loss mechanisms. In addition to the oscillation, a relaxing component (A_{fast} with depolarization rate λ_{fast}) is observed. Even though the depolarization of the muonium state is affected by the cobalt stray field, measuring at each implantation energy in the separate voltage states allows us to fix to the average μ^{+}@C_{60} asymmetry A, phase angle φ and slow depolarization rates $\lambda_{\mu+@C60}$. This allows us to fit the average frequency of the muonium states and the corresponding depolarization rate ($v_{\mu+@C60}$ and $\lambda_{\mu+@C60}$, respectively). The result of using this single oscillation model can be seen in figure S12C. This fit averages all the frequencies in the charged and neutral molecules. It demonstrates that, although the uncertainty is quite large, even averaging for all molecular states the only statistically significant change occurs at the stopping energies (10 and 12 keV) for the MnO_X/C_{60}, where we observe a 13±8% and 26±9% increase in frequency respectively –Fig. S12.

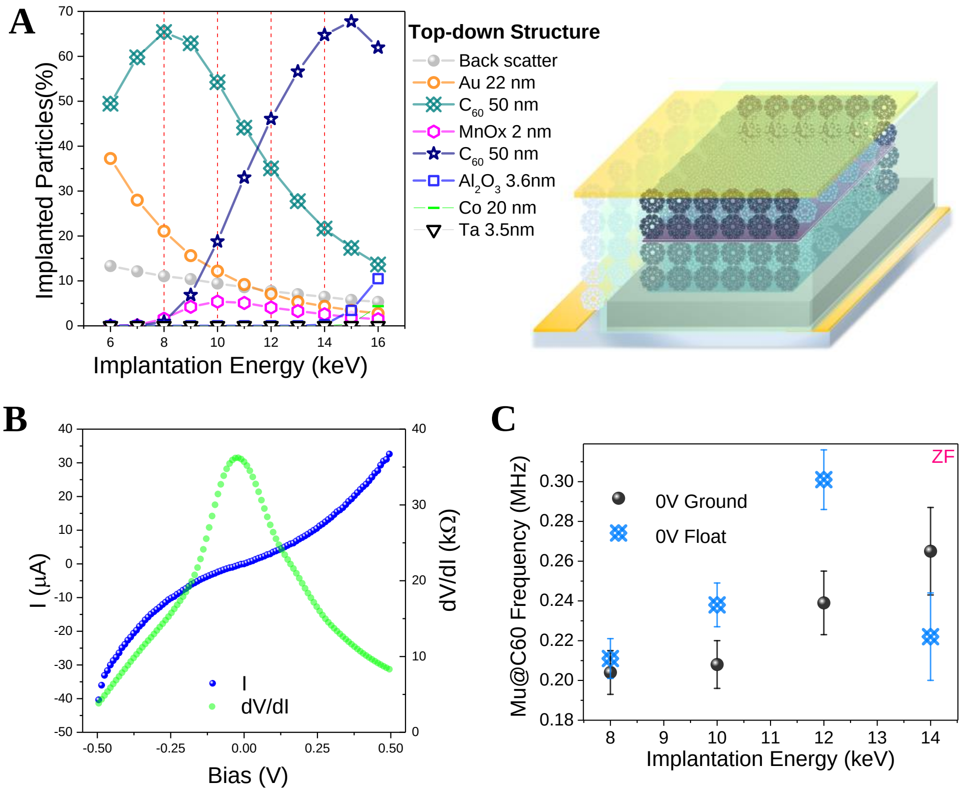

Fig. S12. LEM experimental details for electrically charged sample. (A) Left: Energy dependence of the number of muons stopping in each layer of the fabricated wide area molecular junction. Right: The structure of the device is shown next to a schematic of the finished device. **(B)** Room temperature electron transport characteristics of the wide area junction shows a non-linear dependence of the resistance with voltage as would be expected for tunneling across the Al_2O_3 barrier. **(C)** One-frequency fit of all the muonium states (μ^+@C_{60} and μ^+@C_{60}[-]) to a single oscillation frequency in zero field and with the device in the ground or floating states (after a 0.2 V bias being applied).

As previously discussed, we expect to observe the onset of a second muonium frequency for muonium in spin polarized C_{60}. We therefore used a two frequency model for the ZF data as

$$A_s(t) = A_{fast}e^{-\lambda_{fast}*t} + \sum_{i}^{2} A_i\cos(\varphi + 2\pi v_i t)e^{-\lambda_i * t}$$

Here A_i accounts for the typical anisotropic μ^+@C_{60} state with a frequency of 0.2-0.4, and also for a second, higher frequency oscillation that we attribute to spin polarized charged C_{60} (μ^+@C_{60}[-]). The results obtained by this model are shown in the main manuscript and Fig.S13. This model faces challenges from the increase in free parameters. On the other hand, rather than averaging over all the C_{60} molecules for the penetration profile of the muons, the model can now fit separately those molecules that are charged and therefore may become spin polarized. For the electrically biased sample [Figs. 4A-C in the manuscript], the onset of an emergent frequency is indeed detected by this model at the 10 and 12 keV implantation energies. For these depths, systematic fitting shows that what is effectively an almost flat ($f < 0.05$ MHz, $\lambda = 0\ \mu s^{-1}$) background function, with small asymmetry, picks up a 0.19±0.01 and 0.28±0.03 MHz oscillation frequency in the floating state. In the charged state, the emergent oscillation remains constant at 8 and 14 keV energies, further away from the MnOx/C_{60} interface. The emergent muonium oscillations at 10 and 12 keV possess respective depolarization rates of 0.6±0.2 and 1±0.3 μs^{-1}, demonstrating a dramatic change in the magnetic field distribution at these implantation depths after the application of a voltage.

In a transverse magnetic field (perpendicular to the muon polarization, but parallel to the plane of the sample), we need only to model precession of the μ^+ in some static magnetic field, $\overline{B}_{local}$. The TF-μSR data is therefore fitted with the function

$$A_s(t) = A\cos\left(\gamma_\mu \overline{B}_{local} t + \varphi\right)e^{-\lambda t}$$

With $\gamma_\mu = 2\pi * 135.5$ MHz T^{-1} being the muon gyromagnetic ratio. Once again λ accounts for depolarization mechanisms of the μ^+, particularly due to inhomogeneous magnetic field distributions –see Figures S13C-D below for the electrically biased sample and Figure 4F in the manuscript for the photovoltaic sample.

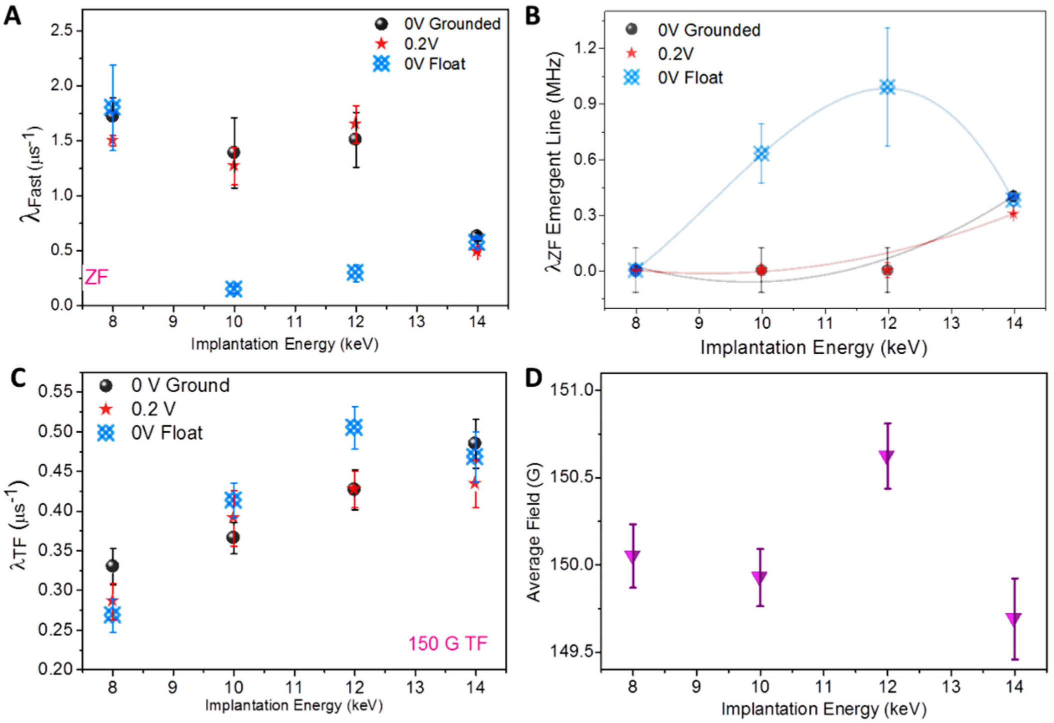

Fig. S13. Fitting of LEM data for electrically charged sample. Parameters obtained by use of the multiple muonium frequency model. **(A)** The energy and voltage dependence of the fast depolarization and associated asymmetry are seen to change at 10 and 12 keV. **(B)** The energy and voltage dependence of λ associated with the emergent C_{60} muonium state shows a dramatic increase for 10 and 12 keV after a bias has been applied. **(C)** With an external magnetic field in the plane of the sample being applied transversal (TF) to the majority muon spins, we measure an increase in the depolarization rate of the TF-μ^+ precession signal from 0.43±0.03 in the ground state to 0.51±0.03 μs^{-1} in the floating state at 12 keV and from 0.37±0.02 to 0.41±0.02 μs^{-1} at 10 keV. **(D)** Local field averaged over the different electrical states, with a maximum at the MnO_x/C_{60} interface.

The optical effects measured in the LEM photovoltaic sample at 250 K (Figs. 4D-F in the manuscript) is also measured at 50 K with a similar magnitude, Fig. S14.

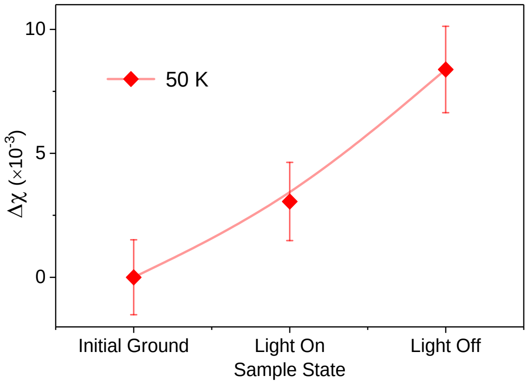

Fig. S14. LE-μSR probing of a photovoltaic sample at 50 K in a transverse field of 300 G. As it was the case at 250 K, there is an increase in the susceptibility under light irradiation and in the floating state. However, the change under light irradiation is smaller (and within uncertainty of the initial ground) than at 250 K. This could be due to the approx. 10 times smaller photocurrent flow at 50 K resulting in longer charging time.

C_{60} compounds may display magnetic order –e.g. EDTA-C_{60} is ferromagnetic at low temperatures, and C_{60}-O may show paramagnetic or superparamagnetic behavior. Figures S15 below shows that the combination of both materials (C_{60} and MnO_2) appears to enhance the magnetic signal, in particular when the interface has been charged by exposure to light -the half-metallic properties of the MnO_2 surface extend to the C_{60}, which is further charged by oxygen hybridization at the interface. The moments observed in Fig. S15a are very weak and therefore highly sensitive to contaminants etc. Fig. S15b shows how the moments may become larger once the full device is grown and the interface charged via light exposure, the magnetic properties are further enhanced –although now there is a large background due to the cobalt electrode and its interaction with the C_{60} layer (*13*).

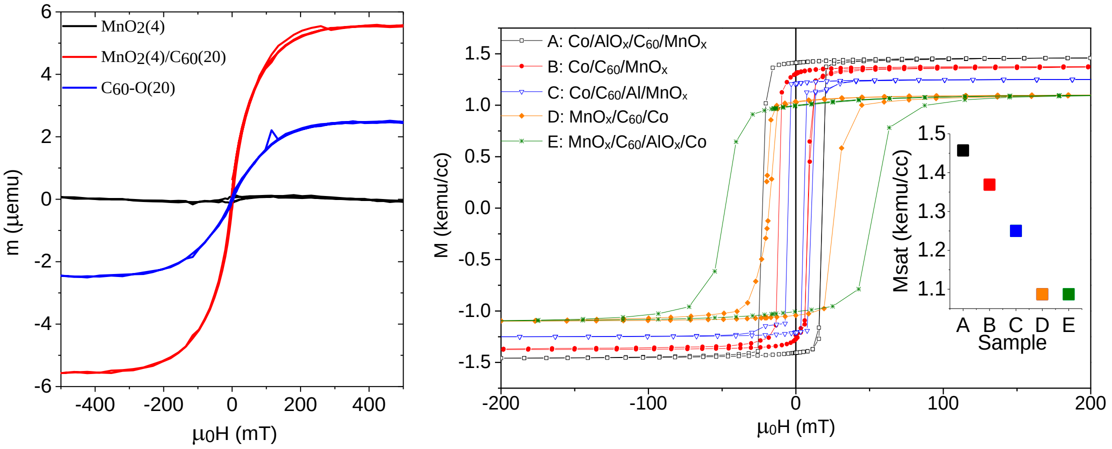

Fig. S15. Magnetometry data. Left: Magnetometry of C_{60} oxide and MnO_x with and without C_{60}. Right@ Magnetometry of Co/C_{60}/MnO_x multilayers with different configurations. Decoupling the C_{60} and MnO_x (sample C) or reversing the structure (samples D and E) leads to lower magnetization.
